# Special Hospitals: Ophthalmic and Miscellaneous

**Published:** 1892-10-15

**Authors:** 


### The Institutional Workshop.

### HOSPITAL ADMINISTRATION.

### V.—SPECIAL HOSPITALS: OPHTHALMIC AND MISCELLANEOUS. COST OF MANAGEMENT.*

#### Ophthalmic Hospitals.

Many have contended that at the present day when so much attention is devoted to diseases of the eye at general hospitals, most of which have special ophthalmic departments with a considerable number of beds, it would be well to discontinue this group of special hospitals altogether. Mr. Brudenell Carter, himself an ophthalmic surgeon of eminence, who is a member of the staff of St. George's Hospital, and who was formerly surgeon to the Royal South London Ophthalmic Hospital, holds this opinion now very strongly. It is reasonable, however, to conclude, when all the circumstances are fairly considered, that the ophthalmic hospital fulfils a useful purpose, and that its continuance is desirable in the public interest. Patients suffering from eye diseases very frequently enjoy good bodily health, and in consequence they are able to be up and about during the greater part of each day. If their bodily health is to be maintained it is desirable that a large amount of day room space should be devoted to them, which it would be difficult to supply in the arrangement of a general hospital. We have given much consideration to the question, and after visiting the ophthalmic hospitals and the special departments connected with general hospitals, we are of opinion that it is desirable to continue, if not to extend, the former institutions. There are five metropolitan ophthalmic hospitals, all of which are contained in the following list:—

Metropolitan Ophthalmic Hospitals.

| Name of Hospital. | Beds for Occupation. | Average Number Occupied. | Total In-Patients Admitted. | Total Out-Patients Admitted. | Percentage of Cost of Administration to that of Maintenance. |
|---|---|---|---|---|---|
| Royal London, Moorfields | 100 | 80 | 1,892 | 2,558 | 12·142 |
| Royal South London ...... | reb | uild | ing | 6,876 | 25·120 |
| Royal Westminster ...... | 30 | 25 | 405 | 8,985 | 25·261 |
| Western Ophthalmic......... | 18 | 2 | 33 | 2,420 | 27·380 |
| Central London ............... | 13 | 5 | 157 | 8,195 | 21·114 |

The figures given in this table are not, on the whole, satisfactory. It is difficult to understand—due regard being paid to economical considerations—how it comes to pass that the most efficient of our ophthalmic hospitals—the Royal London—is able to maintain the high state of excellence to which it has been brought by an expenditure of little more than 12 per cent. upon management, whilst the other similar institutions expend upon management sums varying from 27 to 21 per cent. This difference is so large as to call for strong protest, and it is certainly most unsatisfactory that a hospital like the Western, which only had an average of two beds occupied, and which treated but 2,420 out-patients, should have expended over 27 per cent. upon management. We should like to hear the reasons which the managers of four out of the five ophthalmic hospitals can adduce in justification of the very large expenditure upon management, and to know how they account for the comparatively small expenditure at the Royal London, Moorfields.

* Previous articles of this series have appeared in The Hospital, page 355, for August 27th, page 381 for September 3rd, page 13 for October 1st, and page 31 for October 8th.

#### Miscellaneous Hospitals.

Besides the various groups of special hospitals which we have dealt with in previous articles, there remain twenty others which it is difficult to classify. We shall, therefore, group them as "miscellaneous," and divide them into two lists, viz.: (*a*) those with 100 beds and upwards, and (*b*) those with 50 beds and under. This is anything but an ideal arrangement, but it is the one which seems most feasible, and so we adopt it. It will be seen from the following list that there are five miscellaneous hospitals, containing 100 beds and upwards:—

(*a*) Large Miscellaneous Metropolitan Hospitals.

| Name of Hospital. | Beds for Occupation. | Average Number Occupied. | Total In-Patients Admitted. | Total Out-Patients Admitted. | Percentage of Cost of Administration to that of Maintenance. |
|---|---|---|---|---|---|
| London Fever...... ........... | 200 | 57 | 515 | none. | 25·113 |
| National for the Paralysed and Epileptic ............... | 170 | 156 | 839 | 3,938 | 13 861 |
| Female Lock ................... | 135 | 96 | 647 | none. | 16·194 |
| Cancer, Brompton............ | 105 | 76 | 682 | 1,106 | 18·033 |
| London Temperance ......... | 100 | 62 | 751 | 5,462 | 5·538 |

None of the hospitals included in the table, except the first, present any special features which warrant an expenditure upon management greater than that laid down for general hospitals, viz., 15 per cent. The London Fever Hospital has to be maintained continuously in an efficient condition ready to admit patients, although few fever cases may prevail in London, and so the annual expenditure upon maintenance in a given year may appear altogether out of proportion to that upon administration. The outlay on administration at all fever hospitals is necessarily large and continuous, though the expenditure upon maintenance may be comparatively small during any given year. 25 113 per cent. is the cost of administration to that of maintenance at the London Fever Hospital during 1891, but these figures are considerably modified by the fact that the demand upon the beds was so small that only 57 out of 200 were in daily occupaton. These figures, therefore, explain themselves. The London Temperance Hospital, again, is for all practical purposes a general hospital, and it is highly creditable to the authorities that only 5½ per cent. should be expended upon the management. There does not appear to be any good or sufficient reason why the expenditure upon management at the Cancer Hospital should amount to 18 per cent., or that at the Female Lock to upwards of 16 per cent., and we should hope that those concerned will take steps to reduce this expenditure to a maximum of 15 per cent. The National Hospital for the Paralysed and Epileptic is one of the best administered of metropolitan institutions, and it is highly creditable to the authorities—having regard to the special difficulties they have to face in raising income —that the expenditure on management should be less than 14 per cent. There can be no question that in this country we have far too few beds available for cases of paralysis and epilepsy, and we could wish some wealthy philanthropist would communicate with Mr. Burford Rawlings, the able director of the National Hospital, with a view to placing at his disposal many thousands of pounds to enable him to take proper steps to increase the accommodation for these cases, and to develop his institution still further. The most ardent advocate of special hospitals who regards lavish expenditure upon management as a detail must be forced to admit in all fairness that if Mr. Rawlings is able to maintain the high state of efficiency which prevails at the National Hospital by an expenditure of less than 14 per cent. upon management, there is no reason why other special hospitals should not reduce their expenditure so as to bring it into line with that of one of the ablest of London hospital secretaries. The results achieved at the National Hospital prove beyond dispute that 15 per cent. is the maximum sum which ought to be spent upon management by any of the hospitals included in the above table, with the exception of the London Fever Hospital, for the special reasons already given.

### (b) Smaller Miscellaneous Metropolitan Hospitals.

We now come to the last group of special hospitals, a very miscellaneous one, which embraces all the institutions not included in the previous articles and tables, none of which contain more than 50 beds. Let us first give our table:—

(b) Smaller Miscellaneous Metropolitan Hospitals.

| Name of Hospital. | Beds for Occupation. | Average Number Occupied. | Total In-Patients Admitted. | Total Out-Patients Admitted. | Percentage of Cost of Administration to that of Maintenance. |
|---|---|---|---|---|---|
| Royal Orthopædic | 50 | 50 | 154 | 1,032 | 21·968 |
| National Orthopædic | 35 | 34 | 127 | 710 | 20·179 |
| St. Mark's, for Fistula | 34 | 21 | 311 | 843 | 23·329 |
| City Orthopædic | 27 | 18 | 150 | 2,000 | 26·777 |
| St. Peter's, for Stone | 26 | 15 | 367 | 4,420 | 20·593 |
| Throat Hospital, Golden Square | 26 | 18 | 526 | 7,260 | 10·947 |
| National Heart and Paralysis | 25 | 23 | 126 | 1,769 | 24·017 |
| Epilepsy and Paralysis, Regent's Park | 25 | 19 | 103 | 708 | 23·142 |
| Male Lock | 20 | 13 | 264 | 3,822 | 11·015 |
| Central London Throat and Ear | 16 | 12 | 228 | 6,675 | 12·010 |
| Gordon, for Fistula | 11 | 8 | 123 | 498 | 14·963 |
| Stamford Street Skin | 10 | 1 | 12 | 5,443 | 16·370 |
| Royal Ear | 9 | 5 | 153 | 2,742 | 14·531 |
| London Dental | — | — | — | 28,105 | 24·455 |
| National Dental | — | — | — | 23 197 | 24·834 |

With every desire to do justice, in the most liberal spirit, to the smallest special hospital, we are bound to say that in our judgment in none of these institutions ought the percentage of cost of administration to that of maintenance to exceed 20 per cent. The foregoing table shows that there is much room for improvement so far as the majority of the hospitals it contains are concerned. The Throat hospitals are entitled to the highest praise, seeing that the percentage of administration at the Central London only amounts to 12 per cent., whilst at the Golden Square Hospital it is less than 11 per cent. These figures justify the existence and the continuance of these institutions, and should encourage the public to provide the managers with all the funds they may need to adequately carry out the useful work they undertake. How is it, we wonder, that the expenses at the Male Lock only amount to 11 per cent., whereas at the Female Lock they exceed 16 per cent.? It is creditable to the authorities of the Royal Ear, and the Gordon Hospital for Fistula, that they should spend less than 15 per cent. upon administration, and to the Stamford Street Skin Hospital that less than 16½ per cent. is spent upon management. We hope the giving public will take careful note of these figures when they next distribute their gifts among the hospitals. Any philanthropist is wise to encourage economy in administration, and the special articles we have published on the metropolitan hospitals ought to prove serviceable by enabling all who desire to spend their alms wisely, to secure that the greater proportion of their gifts shall go to relieve the sufferers whom they desire to benefit.

